# Development and validation of a nomogram model for predicting unplanned readmission in patients with acute pancreatitis

**DOI:** 10.3389/fendo.2026.1764742

**Published:** 2026-03-11

**Authors:** Ping Zhu, Weiping Fang, Huifang Tu

**Affiliations:** 1 Department of Gastroenterology, The First People’s Hospital of Linping District, Hangzhou, Zhejiang, China; 2 Department of Gastroenterology, The First People’s Hospital of Jiande, Hangzhou, Zhejiang, China

**Keywords:** acute pancreatitis, nomogram, predicting, readmission, unplanned

## Abstract

**Objective:**

The objective of this study was to develop and validate a nomogram for predicting 1-year unplanned readmission in patients with acute pancreatitis (AP) to identify high-risk populations.

**Methods:**

We retrospectively selected 474 AP patients who were treated and discharged from the First People’s Hospital of Linping District, Hangzhou City, from 1 January 2021 to 31 December 2023. These patients were randomly divided into a training cohort (*n* = 332) and an internal validation cohort (*n* = 142) in a 7:3 ratio. In addition, 218 AP patients treated during the same period at the People’s Hospital of Jiande City were selected as an external validation cohort. The least absolute shrinkage and selection operator (LASSO) was used for variable selection, and multivariable logistic regression was applied for model development. A nomogram was then constructed to estimate the risk of 1-year unplanned readmission. Model performance was evaluated using the consistency index (C-index), calibration curves, receiver operating characteristic (ROC) curves, and decision curve analysis (DCA).

**Results:**

Within 1 year, the incidence of unplanned readmission was 36.1% (120/332) in the training cohort, 40.1% (57/142) in the internal validation cohort, and 42.7% (93/218) in the external validation cohort. Six independent predictors of unplanned readmission in patients with AP were identified, including biliary AP, diabetes, alcohol, infected pancreatic necrosis (IPN) at first admission, acute peripancreatic fluid collection (APFC), and readmission score. The nomogram demonstrated sufficient predictive accuracy, with area under the curve (AUC) values of 0.739 (95% confidence interval [CI]: 0.684–0.794), 0.836 (95% CI: 0.770–0.902), and 0.704 (95% CI: 0.636–0.772) in the training cohort, internal validation cohort, and external validation cohort, respectively. The calibration curve showed good agreement between the predicted risk and the actual risk observed.

**Conclusions:**

The nomogram developed in this study demonstrates good predictive value for unplanned readmission in patients with AP and may help identify high-risk populations.

## Introduction

Acute pancreatitis (AP) is a systemic disease caused by inflammatory lesions of the pancreas. Its main clinical manifestation is abnormal activation of pancreatic digestive enzymes, leading to local and systemic inflammatory reactions in pancreatic tissue. AP is prone to multiple complications, and critically ill patients with multiple organ failure have a high mortality rate (1, 2). The treatment principles of AP include supportive therapy, prevention of physical weakness and malnutrition, and alleviation of recurrent AP (3). Although most AP patients recover in the short term after standardized treatment, the unplanned readmission rate ranges from 7% to 34% (4, 5). Readmission not only increases the physical and medical burden for AP patients but is also closely associated with serious complications such as infected pancreatic necrosis and multiple organ failure (1–5). Therefore, accurately identifying high-risk groups for readmission and developing targeted prevention strategies are key to reducing readmission in AP patients.

At present, scoring systems such as Acute Physiology and Chronic Health Evaluation (APACHE) II score, Ranson score, and BISAP score are commonly used to predict readmission in AP (4–7). Although these scoring systems are applied in clinical practice, they have limitations, including poor predictive performance and cumbersome evaluation methods (5–7). In addition, Whitlock et al. (8) reported five variables independently associated with readmission at discharge: gastrointestinal symptoms, solid diet intolerance, presence of pancreatic necrosis, antibiotic treatment, and persistent pain. Using these five variables, a score was developed to classify patients into three risk levels for readmission: low risk (≤ 1 point, 4%–5% readmission rate), medium risk (2–3 points, 15%–18%), and high risk (≥ 4 points, 68%–87%). The nomogram prediction model is a visualization tool that integrates multiple predictive factors to estimate the probability of an individual experiencing a specific event. It is simple and convenient to use (9, 10). The 30-day readmission nomogram developed by Ding et al. (10) performed well in predicting readmission in AP. The shorter time span limits their ability to provide consistent clinical guidance. AP patients, however, may experience persistent organ failure during the onset of the disease, with a prolonged treatment course, complex and variable conditions, and a poor prognosis (9–11). Nieto et al. (12) reported an 11-month readmission rate of 43.1% for patients with alcoholic AP. To further understand the extent of unplanned readmission of patients after discharge, this study investigated the incidence and independent risk factors of unplanned readmission in AP patients within 1 year.

## Materials and methods

### Patients

AP patients who received treatment and were discharged from the First People’s Hospital of Linping District, Hangzhou City, from 1 January 2021 to 31 December 2023 were included in the study. The diagnosis of AP required two of the following three characteristics (13): (1) upper abdominal pain; (2) serum lipase or amylase activity at least three times the normal upper limit; (3) characteristic manifestations of AP on computed tomography (CT) or magnetic resonance imaging. Patients met the clinical discharge criteria if they had no fever, no abdominal pain symptoms, no systemic inflammatory response syndrome (SIRS), and no organ failure. Exclusion criteria include: (1) age < 18 years old; (2) patients who died during hospitalization; (3) patients with complications of chronic pancreatitis or pancreatic tumors; (4) patients who were pregnant. Patients meeting the inclusion criteria were randomly assigned to the training cohort and internal validation cohort in a 7:3 ratio. In addition, AP patient data from the First People’s Hospital of Jiande City during the same period were collected as an external validation cohort.

### Data collection and definition

The primary outcome was unplanned readmission within 1 year after discharge. Unplanned readmission was defined as an unexpected readmission related to acute pancreatitis or its complications. Patients who had no unplanned readmission within 1 year after discharge were classified as having no event. Planned or elective readmissions for scheduled procedures or examinations were excluded and coded as “no unplanned readmission”, including readmissions for elective cholecystectomy, scheduled removal or management of drainage or enteral nutrition tubes, and planned stent exchange or removal. Readmissions triggered by symptom recurrence or clinical deterioration were classified as unplanned readmissions, even if procedures or examinations were performed during that hospitalization. Among 706 eligible patients entering follow-up, 14 (2.0%) were lost to follow-up and excluded because their 1-year readmission status could not be determined, leaving 692 patients for model development and validation.

The collected risk factors include demographic characteristics and clinical data. The highest value of laboratory examination indicators within 24 h of patient admission was selected. Demographic characteristics include gender, age at diagnosis, body mass index (BMI), smoking, and alcohol use. In this study, alcohol refers to a documented history of regular alcohol use recorded in the admission history and/or discharge records and was coded as a binary variable (yes/no). Alcohol intake dose was not further analyzed because dose information was not consistently recorded in routine electronic medical records across all patients.

The following clinical data were collected for each patient and used for analysis:

1. Etiology of AP: includes biliary, hyperlipidemia, alcoholic, other, and mixed causes. Biliary pancreatitis refers to pancreatitis caused by various biliary diseases. Hyperlipidemic pancreatitis is diagnosed when the serum triglyceride level is ≥ 11.3 mmol/L or between 5.65 and 11.3 mmol/L (500–1000 mg/dL), after ruling out other causes. Alcoholic pancreatitis is defined as drinking alcohol for more than 5 years with an average daily alcohol intake exceeding 50 g. Drug-induced pancreatitis, endoscopic retrograde cholangiopancreatography (ERCP)-related pancreatitis, traumatic pancreatitis, autoimmune pancreatitis, pregnancy-associated pancreatitis, and idiopathic pancreatitis are classified as “other causes”. Patients with two or more etiologies are classified as “mixed etiologies”.
2. Past history: includes hypertension, diabetes, fatty liver, hyperlipidemia, and AP history. Diabetes was defined as a pre-existing diagnosis documented in the medical history and/or discharge diagnosis, and/or the use of glucose-lowering medication during hospitalization, and was coded as a binary variable (yes/no). Diabetes type and glycemic control status were not further categorized because these data were not uniformly available for all patients in this retrospective cohort.
3. Complications: include acute respiratory distress syndrome (ARDS), septic shock, pseudocyst, infected pancreatic necrosis (IPN), and acute peripancreatic fluid collection (APFC).
4. Laboratory examination indicators: include hematocrit, potassium, blood calcium, total bilirubin, urea nitrogen, creatinine, cholesterol, and C-reactive protein.
5. Hospitalization and discharge data: includes hospitalization duration, nutritional support method, and discharge score. The discharge score was assessed using the scoring system proposed by Whitlock et al. (8). Relevant indicators within 24 h before discharge were used to predict 30-day readmission of AP patients. These indicators include insufficient diet at discharge (three points), gastrointestinal symptoms at discharge, including nausea, vomiting, or diarrhea (three points), pancreatic necrosis at discharge (two points), use of antibiotics at discharge (two points), and pain at discharge (one point).

The data were collected by trained medical personnel involved in the study. Follow-up was conducted up to 1 year after discharge for each patient.

The data sources included direct communication, telephone consultation, and electronic medical records. Readmission status within 1 year after discharge was ascertained primarily by review of electronic medical records and supplemented by WeChat or telephone contact when necessary. For participants who could not be reached, at least three contact attempts were made on different days and at different times. Loss to follow-up was defined as failure to ascertain the 1-year readmission status after these efforts.

### Statistical analysis

All statistical analyses were performed using SPSS version 26.0 (IBM Corp., Armonk, NY, USA) and R software version 4.0.2 (R Foundation for Statistical Computing, Vienna, Austria). Normality of continuous variables was assessed using the Shapiro–Wilk test. Nonnormally distributed variables are presented as median (interquartile range) and were compared using the Wilcoxon rank-sum test. Categorical variables are presented as *n* (%), and the Chi-square test was used. Missing data were infrequent in the candidate predictors (overall missingness of approximately 2.7% and maximum missingness for any single variable < 8%). To reduce potential bias and avoid loss of statistical power, missing values were handled using multiple imputation by chained equations (MICE). Five imputed datasets (*m* = 5) were generated, and estimates were pooled using Rubin’s rules. The imputation model included all candidate predictors and the outcome indicator, with continuous and categorical variables imputed using appropriate regression models within the MICE procedure. Least absolute shrinkage and selection operator (LASSO) regression was used for variable selection. Continuous predictors were standardized prior to LASSO. The penalty parameter (λ) was selected using 10-fold crossvalidation based on binomial deviance, and λ was chosen according to the 1-standard-error rule (λ_1se) to obtain a parsimonious model with improved stability. Predictors with nonzero coefficients at the selected λ were retained for subsequent multivariable logistic regression and nomogram construction. The predictor variables selected by LASSO regression were then merged into logistic regression to construct a nomogram prediction model for unplanned readmission of AP patients within 1 year. The sample size was considered adequate for prediction modeling based on the events-per-variable principle. The final model included six predictors, and the training cohort contained 120 readmission events, yielding an events-per-variable value of 20, which supports stable coefficient estimation and reduces the risk of overfitting. The performance of the prediction model was evaluated in the training cohort, internal validation cohort, and external validation cohort. Receiver operating characteristic (ROC) curves and calibration curves were used to evaluate model discrimination and calibration, respectively. For reporting sensitivity and specificity, the optimal probability threshold was determined in the training cohort by maximizing the Youden index on the ROC curve. The same threshold was applied to the internal and external validation cohorts to derive sensitivity, specificity, positive predictive value, and negative predictive value. The area under the curve (AUC) of the ROC curve ranges from 0.50 to 1.00, with values closer to one indicating better prediction performance. The calibration process assesses whether the predicted risks are consistent with the observed risks. To evaluate the clinical practicality of the model, decision curve analysis (DCA) was used to assess its effectiveness. A *p* < 0.05 indicates statistical significance.

## Results

Among 706 eligible patients initially entering follow-up, 14 (2.0%) were lost to follow-up and excluded due to unknown 1-year readmission status, resulting in 692 patients included in the final analysis. A total of 474 eligible AP patients were collected from the First People’s Hospital of Linping District, Hangzhou. Randomly divided into two cohorts in a 7:3 ratio, 332 patients were assigned to the training cohort and 142 patients to the internal validation cohort. The external validation cohort includes 218 AP patients from the First People’s Hospital of Jiande. The clinical characteristics of the patient are shown in **Table 1**. During the follow-up period, the unplanned readmission rates for the training cohort, internal validation cohort, and external validation cohort were 36.1% (120/332), 40.1% (57/142), and 42.7% (93/218), respectively. A total of 270 patients experienced unplanned readmission, corresponding to an overall incidence of 39.0% (270/692). Abdominal pain (66.3%) was the most common symptom during unplanned readmission, followed by nausea and vomiting (13.7%). Suspected infection readmission (7.4%) and feeding tube complications (3.7%) were also common reasons for readmission. In addition, 8.9% of patients had two or more of the above symptoms (**Supplementary Table 1**). A total of 58.9% (159/270) of patients were readmitted within 1 month, 25.9% (70/270) were readmitted within 1–3 months, and 15.2% (41/270) were readmitted within 3–12 months (**Supplementary Table 1**).

**Table 1:** Basic characteristics and clinical overview of patients.

Variables	Training (*N* = 332)	Internal validation (*N* = 142)	*p*-value^a^	External validation (*N* = 218)	*p*-value^b^
Male (yes), *n* (%)	172 (51.8)	69 (48.6)	0.521	121 (55.5)	0.395
Age (years), M (IQR)	48 (39–60)	45.5 (40–57)	0.949	47 (42–56)	0.861
BMI (kg/m^2^), M (IQR)	22.6 (21.1–25.05)	23.5 (20.5–26.3)	0.156	23.4 (21.2–25.5)	0.151
Biliary (yes); *n* (%)	141 (42.5)	52 (36.6)	0.235	111 (50.9)	0.052
Hypertension (yes), *n* (%)	184 (55.4)	66 (46.5)	0.074	140 (64.2)	0.040*
Diabetes (yes), *n* (%)	131 (39.5)	44 (31.0)	0.080	100 (45.9)	0.136
Fatty liver (yes), *n* (%)	114 (34.3)	54 (38.0)	0.442	66 (30.3)	0.321
Hyperlipidemia (yes), *n* (%)	142 (42.8)	69 (48.6)	0.243	100 (45.9)	0.474
Severity of AP					
Mild	90 (27.1)	42 (29.6)	0.278	58 (26.6)	0.417
Moderate and severe	152 (45.8)	54 (38.0)		90 (41.3)	
Severe	90 (27.1)	46 (32.4)		70 (32.1)	
ARDS (yes), *n* (%)	91 (27.4)	34 (23.9)	0.433	65 (29.8)	0.540
Septic shock (yes), *n* (%)	41 (12.3)	22 (15.5)	0.356	29 (13.3)	0.743
Pseudocyst (yes), *n* (%)	38 (11.4)	14 (9.9)	0.613	23 (10.6)	0.744
IPN (yes), *n* (%)	46 (13.9)	22 (15.5)	0.641	39 (17.9)	0.200
APFC (yes), *n* (%)	111 (33.4)	44 (31.0)	0.603	80 (36.7)	0.432
Smoking (yes), *n* (%)	177 (53.3)	70 (49.3)	0.423	121 (55.5)	0.614
Alcohol (yes), *n* (%)	143 (43.1)	57 (40.1)	0.554	109 (50.0)	0.111
History of AP (yes), *n* (%)	115 (34.6)	53 (37.3)	0.576	87 (39.9)	0.210
Hospitalization time (days)	6 (5–9)	7 (5–9)	0.301	6 (5–9)	0.192
Hematocrit (L/L)	0.38 (0.36–0.43)	0.4 (0.34–0.45)	0.030^*^	0.42 (0.37–0.47)	< 0.001^*^
Blood potassium (mmol/L)	4.01 (3.54–4.54)	3.85 (3.39–4.52)	0.711	4.08 (3.47–4.57)	0.371
Calcium (mmol/L)	2.34 (2.13–2.55)	2.32 (2.06–2.52)	0.399	2.03 (1.86–2.19)	< 0.001^*^
Total bilirubin (μmol/L)	20.05 (11.5–30.5)	17.67 (11.37–33.17)	0.838	15.55 (9.5–26.1)	0.008^*^
Urea nitrogen (μmol/L)	4.67 (3.33–5.81)	4.63 (3.18–5.92)	0.676	5.64 (3.47–7.85)	< 0.001^*^
Creatinine (μmol/L)	78.9 (63.8–101.9)	80.5 (65.98–106.1)	0.497	65.7 (45.5–105.5)	0.001^*^
Total cholesterol (mmol/L)	3.47 (2.26–5.18)	3.41 (1.88–5.3)	0.438	3.24 (1.56–5.43)	0.047^*^
C-reactive protein (mg/L)	108.45 (76–154)	112 (84–152)	0.386	106.8 (82–161.9)	0.443
Nutritional support methods					
Enteral nutrition	184 (55.4)	86 (60.6)	0.579	118 (54.1)	0.650
Parenteral nutrition	75 (22.6)	29 (20.4)		45 (20.6)	
Enteral + parenteral nutrition	73 (22.0)	27 (19.0)		55 (25.3)	
Readmission score	3 (2–5)	3 (2–5)	0.868	4 (2–5)	0.495
Readmission (yes), *n* (%)	120 (36.1)	57 (40.1)	0.410	93 (42.7)	0.126

*AP*, acute pancreatitis; *BMI*, body mass index; *IQR*, interquartile range; *ARDS*, acute respiratory distress syndrome; *IPN*, infected pancreatic necrosis; *APFC*, acute peripancreatic fluid collection; *CRP*, C-reactive protein.

^*^p < 0.05.

a The comparison between the training cohort and the internal validation cohort.

b The comparison between the training cohort and the external validation cohort.

**Figure 1:**
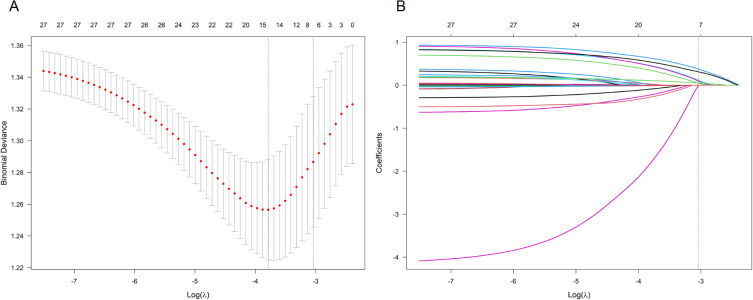
LASSO coefficient profiles for predictors of 1-year unplanned readmission in patients with acute pancreatitis (AP). **(A)** Each curve represents the coefficient profile of a candidate predictor. The *y*-axis indicates the coefficient value, the lower *x*-axis indicates log(λ), and the upper *x*-axis indicates the number of nonzero coefficients. **(B)** Ten-fold crossvalidation was performed to select the optimal penalty parameter λ.

Firstly, we preliminarily selected predictive factors for unplanned readmission through LASSO regression. Continuous predictors were standardized prior to LASSO. Ten-fold crossvalidation was used to select the optimal penalty parameter (**Figure 1**). The selected predictors included biliary etiology, diabetes, alcohol consumption, IPN, APFC, and readmission score. Secondly, six predictive factors were identified as independent risk variables, and a multiple logistic regression model was constructed (**Table 2**). These six predictive factors are biliary etiology (odds ratio [OR]: 2.448, 95% confidence interval [CI]: 1.504–3.985); diabetes (OR: 1.917, 95% CI: 1.173–3.132); drinking alcohol (OR: 1.684, 95% CI: 1.037–2.734); IPN (OR: 2.093, 95% CI: 1.070–4.096); APFC (OR: 1.902, 95% CI: 1.154–3.137); and discharge score (OR: 1.171, 95% CI: 1.039–1.319).

**Table 2:** Multivariable logistic regression analysis of predictors selected by LASSO for nomogram development.

Independent variables	*B*	OR (95% CI)	*p*-value
Biliary	0.895	2.448 (1.504–3.985)	< 0.001
Diabetes	0.651	1.917 (1.173–3.132)	0.009
Alcohol	0.521	1.684 (1.037–2.734)	0.035
IPN	0.739	2.093 (1.070–4.096)	0.031
APFC	0.643	1.902 (1.154–3.137)	0.012
Score at discharge	0.158	1.171 (1.039–1.319)	0.009

*AP*, acute pancreatitis; *IPN*, infected pancreatic necrosis; *APFC*, acute peripancreatic fluid collection; *B*, regression coefficient; *OR*, odds ratio; *CI*, confidence interval.

**Figure 2:**
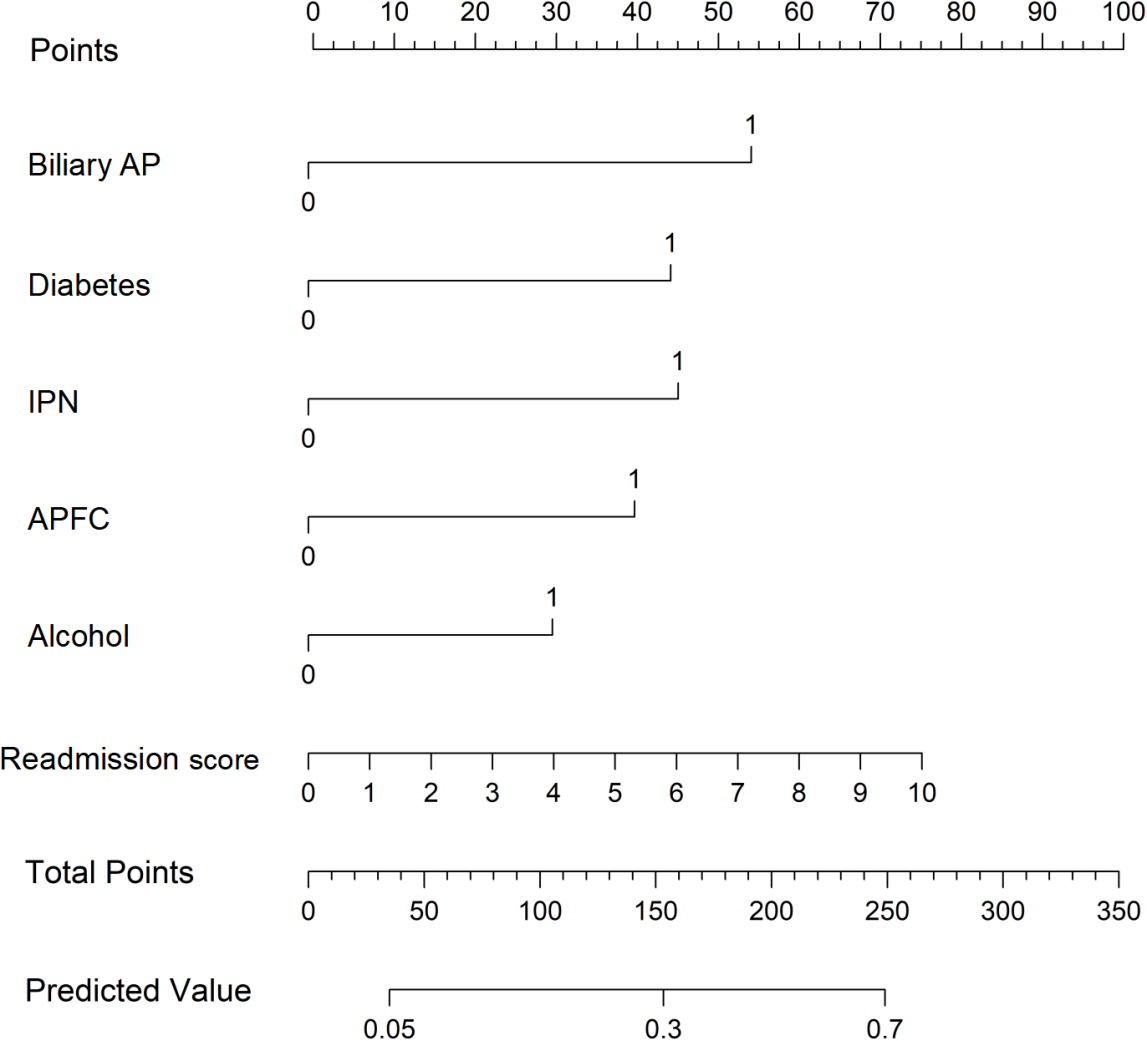
Nomogram for predicting 1-year unplanned readmission in patients with acute pancreatitis (AP). Each predictor level corresponds to a point value. Total points are obtained by summing the points for all predictors, and the total corresponds to the predicted probability of unplanned readmission.

Using R software (version 4.0.2) and its rms package, a nomogram prediction model for unplanned readmission of AP patients was constructed based on the six independent risk factors selected from the multiple logistic regression analysis (**Figure 2**). The sum of the score values corresponding to each predictive indicator in the nomogram was recorded as the total score, and the predicted probability corresponding to the total score represents the risk of readmission. For example, a patient with biliary AP, diabetes, alcohol use, concurrent IPN and APFC, and a readmission score of 4 would have a total score of 242 points (53 + 44 + 45 + 40 + 30 + 30 = 242), corresponding to a predicted probability of unplanned readmission of approximately 68% on the nomogram.

**Figure 3:**
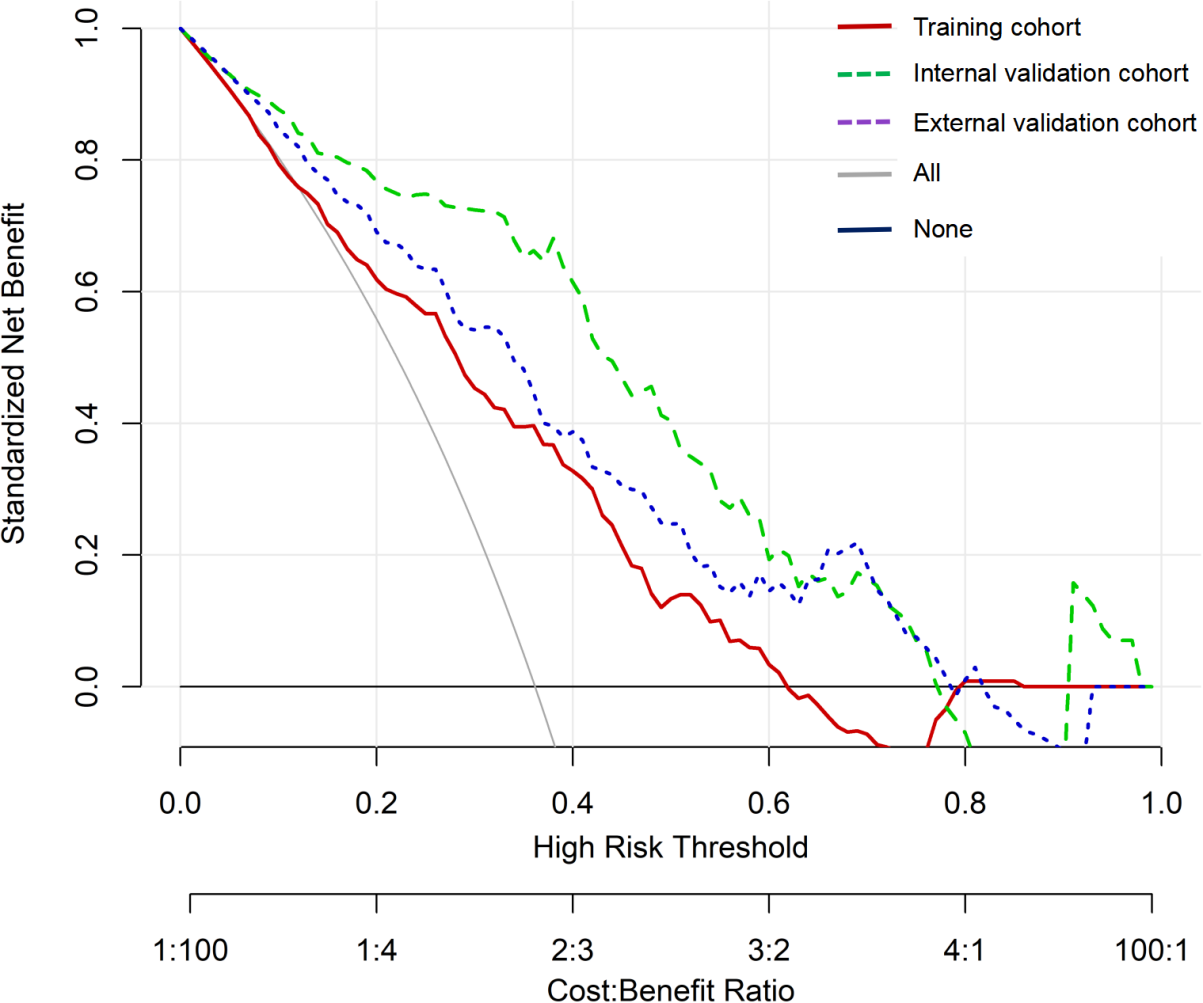
Receiver operating characteristic (ROC) curves and area under the curve (AUC) for the predictive model. ROC, receiver operating characteristic; AUC, area under the curve.

**Figure 4:**
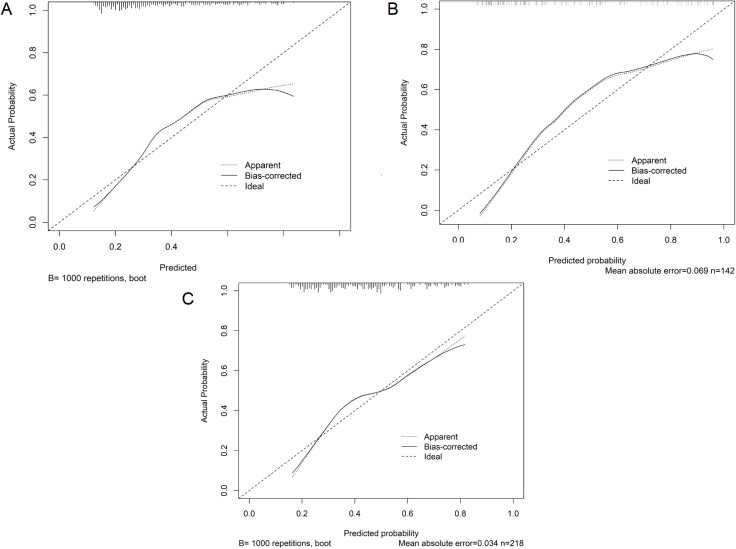
Calibration curves of the prediction model. **(A)** Training cohort. **(B)** Internal validation cohort. **(C)** External validation cohort. The *x*-axis represents the predicted probability of unplanned readmission, and the *y*-axis represents the observed probability of unplanned readmission. The diagonal dashed line indicates perfect calibration, and the solid line represents the performance of the nomogram; closer agreement indicates better calibration.

**Figure 5:**
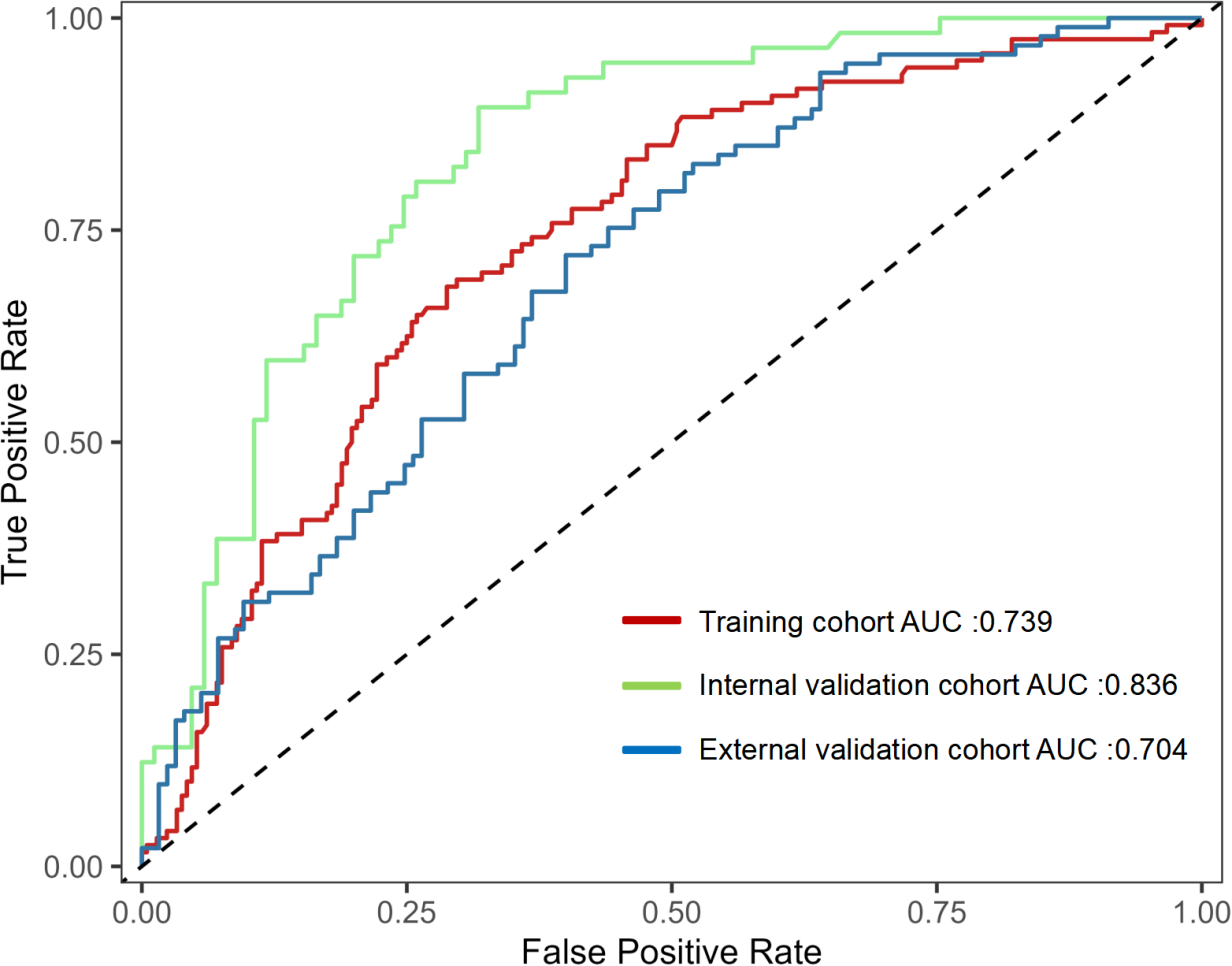
Decision curve analysis (DCA) of the nomogram. DCA, decision curve analysis.

Internal validation of the nomogram model was performed using the Bootstrap method (after 1,000 repeated samplings of the raw data), while external validation was conducted in the external validation cohort. The results showed that the consistency indices (C-indices) of the training cohort, internal validation cohort, and external validation cohort were 0.714 (95% CI: 0.656–0.772), 0.822 (95% CI: 0.763–0.881), and 0.711 (95% CI: 0.652–0.770), respectively. According to the Hosmer–Lemeshow test, the Chi-square values of the training cohort, internal validation cohort, and external validation cohort were 8.642 (*p* = 0.373), 17.215 (*p* = 0.190), and 8.853 (*p* = 0.355), respectively. These results indicate that the predicted outcomes closely match the observed outcomes. The ROC curve in the training cohort showed good discriminative ability (AUC: 0.739; 95% CI: 0.684–0.794; sensitivity = 40.8%, specificity = 84.4%, positive predictive value (PPV) = 59.8%, negative predictive value (NPV) = 71.6%), and the bootstrap-validated C-index (1,000 bootstrap resamples) was 0.773, demonstrating satisfactory predictive performance. The discriminative performance of the model was validated in the internal validation cohort (0.836, 95% CI: 0.770–0.902; sensitivity = 36.8%, specificity = 92.9%, PPV = 77.8%, NPV = 68.7%) and the external validation cohort (0.704, 95% CI: 0.636–0.772; sensitivity = 39.8%, specificity = 80.0%, PPV = 59.7%, NPV = 64.1%) (**Figure 3**). In addition, calibration curve analysis showed good consistency between predicted probabilities and observed readmission rates in the training and validation cohorts (**Figure 4**). DCA indicated that for patients with a threshold probability greater than 0.1, the benefits generated by the nomogram exceeded those of treating all or no patients, demonstrating the nomogram’s clinical utility in predicting readmission (**Figure 5**).

## Discussion

This study included 692 patients with acute pancreatitis from two tertiary centers and evaluated unplanned readmission within 1 year after discharge. A total of 270 patients were readmitted, with an incidence rate of 39.0%, which is comparable to rates reported by Jogi et al. (5) and Nieto et al. (12). Using LASSO selection followed by multivariable logistic regression, six independent predictors of unplanned readmission were identified, and a nomogram incorporating biliary etiology, diabetes, alcohol, infected pancreatic necrosis, acute peripancreatic fluid collection, and readmission score was developed. The model showed moderate discrimination, with AUCs of 0.739 in the training cohort, 0.836 in the internal validation cohort, and 0.704 in the external validation cohort. The calibration curves demonstrated reasonable agreement between predicted and observed risks.

The identified predictors can be interpreted within a clinically coherent framework. Regarding etiology, gallstones remain a leading cause of acute pancreatitis. Patients with biliary etiology accounted for 43.9% (304/692), and the risk of readmission was higher among biliary cases. Biliary pancreatitis may be accompanied by biliary obstruction and cholestasis, which can predispose patients to infectious complications such as cholangitis and liver abscess (1, 2, 14). Infection may further trigger a systemic inflammatory response (14). Consistent with guideline recommendations, the American Gastroenterological Association advises cholecystectomy during the index admission for acute biliary pancreatitis when appropriate (15). Prior studies have reported that index-admission cholecystectomy can reduce short-term readmission rates, although delayed cholecystectomy may be safer in patients with severe inflammation (16, 17).

Metabolic and behavioral factors also contributed to readmission risk. Diabetes has been associated with increased severity and mortality in acute pancreatitis, and our findings were consistent with prior reports, including Ding et al. (10) and Huh et al. (18). An episode of acute pancreatitis is frequently accompanied by stress hyperglycemia or worsening of pre-existing diabetes, often requiring insulin therapy to achieve glycemic control. Persistent hyperglycemia and dysglycemia can impair innate immune responses, including neutrophil chemotaxis, phagocytosis, and microbial killing, thereby increasing susceptibility to infectious complications and potentially contributing to readmission (19). In retrospective data, insulin use should be interpreted cautiously, as it may reflect more severe dysglycemia rather than serving as a direct causal driver of infection risk (20). Insulin therapy is also associated with hypoglycemia. During fasting or gastrointestinal decompression, glycemic variability may be exacerbated and adversely affect recovery (10, 18, 21). Alcohol was another modifiable predictor. Karjula et al. (22) reported high relapse and mortality burdens in alcoholic acute pancreatitis, and mechanistic studies suggest that alcohol and its metabolites can directly injure acinar cells and sustain pancreatic inflammation (22, 23). These findings support targeted interventions on alcohol abstinence as part of postdischarge management (15).

Complications during the index admission and discharge status were strongly associated with readmission. Maatman et al. (24) reported high readmission rates in necrotizing pancreatitis, and our results similarly showed that infected pancreatic necrosis increased readmission risk. IPN is closely related to disease severity and may be accompanied by organ failure and other complications that require prolonged hospitalization and closer postdischarge monitoring (25). Acute peripancreatic fluid collection also increased readmission risk, consistent with findings from Ali et al. (26) and Solakoglu et al. (27). Fluid collections may communicate with the pancreatic duct and contribute to pancreatic fistula formation, ongoing inflammation, and secondary infection, leading to recurrent symptoms and readmission (26, 27). In addition, we incorporated a discharge-related readmission score derived from Whitlock et al. (8), which reflects symptoms and clinical status near discharge. Higher scores were associated with an increased readmission risk in our cohort.

From a clinical perspective, the nomogram is intended to support risk stratification rather than replace clinical judgment. The external validation AUC of 0.704 indicates moderate discrimination, suggesting that the tool may be useful for prioritizing follow-up and tailoring postdischarge management intensity. Decision curve analysis suggested a potential net benefit across a range of threshold probabilities, supporting its possible clinical utility in allocating resources to patients at higher risk. Compared with prior nomogram studies in acute pancreatitis, our work extends the existing evidence by focusing on 1-year unplanned readmission, using readily available variables that capture etiology, comorbidity, complications, and discharge status, and providing external validation across institutions (8–11). These features may facilitate practical implementation in routine care while acknowledging that model transportability can vary across settings.

This study has several limitations. First, it was retrospective in design. Although external validation was performed, prospective multicenter validation is warranted to further evaluate generalizability across different healthcare settings. Second, genetic risk factors were not included. Genetic predisposition may influence host inflammatory responses and susceptibility to recurrent or complicated acute pancreatitis and could provide additional prognostic information for readmission risk beyond routine clinical variables. Third, organ failure-related parameters were not incorporated. Organ dysfunction reflects disease severity and physiological reserve, and incorporating organ failure metrics, particularly dynamic changes during hospitalization, may improve discrimination, calibration, and transportability across settings. These data were not consistently available or standardized in our retrospective electronic medical records, so they were not incorporated into the current model, which may limit model completeness and the achievable predictive performance. Fourth, alcohol exposure was not quantified, and diabetes was not stratified by type or glycemic control status, which may have resulted in residual confounding and limited interpretability and transportability. Fifth, the accuracy of coding in the management database may vary, which could lead to outcome misclassification. Sixth, the duration and frequency of medication use after discharge were not available. Future research should focus on prospective multicenter studies, the incorporation of dynamic or longitudinal indicators, and model recalibration or updating across sites. In addition, integration of the model into electronic medical record systems may facilitate real-time risk stratification and targeted postdischarge follow-up.

## Conclusion

The nomogram model constructed in this study provides an accurate predictive tool for unplanned readmission in patients with AP by integrating etiology, lifestyle habits, underlying diseases, complications, and discharge status. The model demonstrates good discrimination and calibration and performs stably in external cohorts, with high clinical application value. It is recommended that personalized intervention strategies be developed based on the model results in clinical practice to reduce the risk of readmission and improve patient prognosis.

## Data Availability

The original contributions presented in the study are included in the article/**Supplementary Material**. Further inquiries can be directed to the corresponding author.
