# Supplementary material for: Development and validation of a nomogram model for predicting unplanned readmission in patients with acute pancreatitis
Source: Front Endocrinol (Lausanne). 2026 Mar 11;17:1764742. doi: 10.3389/fendo.2026.1764742 (PMC13012988; doi:10.3389/fendo.2026.1764742)
Supplement: Supplementary file 1 [file Table1.docx]

Supplementary Table I Clinical symptoms and time of unplanned readmission of AP patients

| Variables | Training (N=120) | Internal validation (N=57) | External validation (N=93) | Total (N=270) |
| --- | --- | --- | --- | --- |
| Clinical symptoms upon readmission, n (%) |  |  |  |  |
| Abdominal pain | 78 (65.0) | 34 (59.6) | 67 (72.0) | 179 (66.3) |
| Nausea and vomiting | 15 (12.5) | 11 (19.3) | 11 (11.8) | 37 (13.7) |
| Infectious complications | 9 (7.5) | 6 (10.5) | 5 (5.4) | 20 (7.4) |
| Complications of feeding tube | 5 (4.2) | 1 (1.8) | 4 (4.3) | 10 (3.7) |
| Two or more symptom types | 13 (10.8) | 5 (8.8) | 6 (6.5) | 24 (8.9) |
| Time to readmission (months), n (%) |  |  |  |  |
| 1 | 68 (56.7) | 36 (63.2) | 55 (59.2) | 159 (58.9) |
| 1~3 | 35 (29.2) | 16 (28.1) | 19 (20.4) | 70 (25.9) |
| 3~12 | 17 (14.1) | 5 (8.7) | 19 (20.4) | 41 (15.2) |
